# Secure channel estimation model for cognitive radio network physical layer security using two-level shared key authentication

**DOI:** 10.1038/s41598-025-86165-x

**Published:** 2025-01-19

**Authors:** K. Saravanan, K. B. Gurumoorthy, Allwin Devaraj Stalin, Om Prakash Kumar

**Affiliations:** 1 Department of Mechatronics Engineering, KPR Institute of Engineering and Technology, Coimbatore, Tamilnadu 641407 India; 2 Department of Electronics and Communication Engineering, KPR Institute of Engineering and Technology, Coimbatore, Tamilnadu 641407 India; 3 Department of Electronics and Communication Engineering, Francis Xavier Engineering College, Tirunelveli, 627003 India; 4 Department of Electronics and Communication Engineering, Manipal Institute of Technology, Manipal Academy of Higher Education, Manipal, 576104 India

**Keywords:** Cognitive radio networks, CSI, Deep learning, Physical layer security, Shared authentication, Engineering, Electrical and electronic engineering, Mathematics and computing

## Abstract

Physical Layer Security (PLS) in Cognitive Radio Networks (CRN) improves the confidentiality, availability, and integrity of the external communication between the devices/ users. The security models for sensing and beamforming reduce the impact of adversaries such as eavesdroppers in the signal processing layer. To such an extent, this article introduces a Secure Channel Estimation Model (SCEM) using Channel State Information (CSI) and Deep Learning (DL) to improve the PLS. In this proposed model, the CSI is exploited to evaluate the channel utilization and actual capacity availability throughout the allocation intervals. The change in channel capacity and utilization augments the need for security through 2-level key shared authentication. The deep learning algorithm verifies the authentication completeness for maximum channel capacity utilization irrespective of adversary interference. This verification follows mutual authentication between the primary and secondary users sharing the maximum capacity channel with high secrecy. The learning monitors the outage secrecy rates to verify failed allocations such that the replacement for allocation is pursued. Thus, the physical layer security between different user categories is administered through maximum CSI exploitation with high beamforming abilities. The proposed model leverages the secrecy rate by 10.77% and the probability of detection by 15.01% and reduces the interference rate by 11.07% for the varying transmit powers.

## Introduction

Physical layer security is one of the most critical issues in cognitive radio networks (CNRs) because the dynamism of spectrum access extends to questions of sensitive information protection^1^. Unlike the conventional methods of encryption applied at the upper layers, security at the physical layer exploits some inherent properties of the wireless channel. These make sure that the signals cannot be decoded even when they have been intercepted by any unauthorized user or eavesdropper^2,3^. Some of such techniques that critically enhance the security for CRNs are the use of artificial noise generation, beamforming, and exploiting channel state information. These techniques operate at the physical layer and guarantee the delivery of an excellent defense mechanism against a broad spectrum of security threats, like eavesdropping and jamming^4,5^. Physical layer security is pretty important to be part of the CRNs to maintain the user’s privacy and data integrity during data transfer. However, it becomes most crucial in network environments, like spectrum access, which is shared by multiple users^6^.

The CRN involves various security models to ensure the protection of the performance of a network for improving Quality of Service (QoS)^7^. Spectrum availability, user demand, and interference management are some of the major parameters through which QoS in CRNs gets impacted. Given the above factors, QoS/security models have to be embedded in the network design for reliable communication^8,9^. For example, spectrum sensing security models ensure the prevention of attacks from malicious users, which may hinder the accuracy of the spectrum sensing process for the correct detection of the available channels^10^. The authentication models identify the authenticity of the user or the device accessing the network to minimize the risk of unauthorized access^11^. By integrating these security models, it is ensured that QoS is enhanced, along with overall network reliability and efficiency. Focusing on security at different layers of the network, the CRN will be able to provide its users with homogeneous and high-quality service despite the threats^12^.

Security can be made more adaptive and responsive to new threats by embedding learning methodologies into the security framework of a CRN^13^. Techniques from machine learning algorithms like reinforcement and deep learning can be exploited in which security protocols are adapted dynamically according to the present network status^14^. These algorithms analyze patterns in network traffic, user behavior, and spectrum usage to identify the possible security risks and take actions for the optimization of countermeasures. It means that learning-based intrusion detection systems would enable the network to automatically detect and mitigate unauthorized access attempts or jamming attacks, hence improving its resilience^15,16^. The application of learning methods for the optimization of spectrum allocation and sharing strategies guarantees security even in highly dynamic environments for the operation of the network^17^. The article’s contributions are:

(i) To discuss the existing methods for their contribution towards physical layer security in CRNs with specific authentication models disclosed in the past.
(ii) To propose a novel secure channel estimation model aided by congruent deep learning to improve the outage secrecy by reducing the impact of eavesdroppers’ primary and secondary user communications.
(iii) To analyze the proposed model’s performance using secrecy rate, interference, probability of missed detection, time consumption, and probability of detection metrics.
(iv) To verify the proposed model’s performance using a comparative analysis performed with the existing BSE-SSM^31^, JB-IA^33^, and MA-DRL^30^ methods.

Physical layer security in CRN is concerned with secondary user communication. Improper radio resource allocation/ interference due to weak physical layer authentication compromises this communication. For weak/ compromised security a complex key authentication is recommended in conventional methods. Through it administers security eventually the complexity rises behind a saturation point of the number of users. To balance these fundamental changes, more adaptable physical layer security is required for the CRNs. Motivated by this fact, this article proposes a novel; shared key authentication to suppress the aforementioned issues. The organization of the article is: In the following Sect. 2, the related works for CRN physical layer security and authentication are discussed. In Sect. 3, the proposed novel secure channel estimation model is elaborated with suitable illustrations. Section 4 presents the hyperparameter analysis and comparative assessment using different metrics and methods followed by the conclusion and future works in Sect. 5.

## Related works

Khoshafa et al.^18^ proposed a method for improving the security in cognitive radio networks. The approach uses a reconfigurable intelligent surface to enhance the reliability of the secondary network and physical layer security for both the secondary and primary networks. Closed-form expressions for outage probabilities and secrecy capacity for both networks are derived. The method confirms its effectiveness using numerical and simulation results.

Torabi et al.^19^ proposed a secure communication system for cognitive radios. A method for analyzing physical layer security with Alamouti orthogonal space-time block coding and spatially correlated transmit antennas is presented. Closed-form expressions are driven in terms of secrecy metrics, such as strictly positive secrecy capacity, secrecy outage probability, and average secrecy capacity. The method shows how increasing the signal-to-noise ratio will improve security.

Alanazi et al.^20^ designed a secured cognitive radio system based on adaptive power. A method is proposed to compute the Secrecy Outage Probability and the Probability of Strictly Positive Secrecy Capacity via adaptive transmit power along with energy harvesting multiple antennas. The technique considers interference and measures the signal-to-interference-plus-noise ratio at the secondary destination. The approach obtains metrics based on the best antenna configurations at source and destination.

Wu et al.^21^ introduced a secure energy-efficient transmission method. An intelligent reflecting surface is employed to enhance the spectrum efficiency and the physical layer security of cognitive radio networks. The method involves the joint design of beamforming at the base station and the reflecting surface for the maximization of the secrecy energy efficiency. The method substantially improves spectrum efficiency and security by balancing the secrecy rate with the energy consumption.

Ridouani et al.^22^ proposed a secure cooperative cognitive radio network. The approach embeds authentication and shifted spectrum sensing to prevent malicious attacks. Secure compressive sensing is used with a Chebyshev matrix for the detection and removal of malicious users. The method includes a new spectrum sensing approach that senses a wideband spectrum with a high probability of detection and optimum settings.

Giri et al.^23^ proposed a method to identify users as malicious in cognitive radio networks. Extreme learning machines have been used to classify the nature of the users as legitimate or malicious. The approach improves the training time and performance considerably compared to the existing approaches. The method improves the accuracy of classification in managing the security of networks.

Tofiq et al.^24^ proposed a lightweight secure throughput optimization scheme. The approach models the influence of adversary nodes on cooperative spectrum sensing in terms of false alarm and missed detection probabilities. These probabilities are further integrated into the throughput equation of the network to assess its performance. An intrusion detection system is proposed to ensure maximum throughput in the presence of adversary users.

Venkatesan et al.^25^ proposed a method for secure data transmission in cognitive radios. An optimized neural network model is used, called BMHHO–ENN, to detect the attack and classify it. The approach increases security by introducing SHA2-RSA for encrypted communication only when some attack is detected. The technique extracts features from the primary user signal to accurately detect any attacks and works better than the available techniques.

Marriwala et al.^26^ developed an authentication-based approach to prevent attacks. A trust-based security mechanism is followed to authenticate users and mitigate SSDF attacks. The proposed approach validates the authentication framework with the MATLAB simulation results. The method improves spectrum utilization by allowing only authenticated users, who can reduce malicious attack impacts.

Yan et al.^27^ presented a method to improve physical-layer security in cognitive networks. Artificial noise and rate splitting (ANRS) are utilized to enhance transmission secrecy. The primary user generates artificial noise to confuse the eavesdroppers, while the secondary user performs rate splitting. The approach performs better in simulation regarding secrecy performance.

Jiang et al.^28^ developed a secure cognitive multi-user network system. The method addresses the physical layer security in multiuser networks with hardware impairments and channel errors. The technique proposes user scheduling and jammer-aided extensions of the network to improve security. The method derives the closed-form expressions for intercept probability, outage probability, and secrecy throughput.

Liu et al.^29^ designed a security performance analysis of cognitive radio networks. The method involved using IRS-assisted MISO systems for improving confidentiality. The approach optimized beamforming and artificial noise matrices along with phase shifts at IRS for power minimization in the arrangement of secure communication. The method gives the result that the security and reduction in power are improved by increasing transmit antennas and IRS elements.

Lin et al.^30^ proposed a learning-based approach for enhancing security. The optimum resource allocation and secrecy in energy-harvesting cognitive radios are achieved using multi-agent deep reinforcement learning. The approach models the sub-channels and jammers as agents for an optimum power and time allocation strategy. The approach outperforms other existing schemes in terms of secrecy rate and overall performance.

Khanna et al.^31^ proposed a blockchain-based security scheme for cognitive radios. The approach proposed in the paper exploits blockchain technology to allow much-needed improvements in security and spectrum sensing. Adaptive threshold spectrum energy detection is used in the method for identifying and mitigating malicious users. The method improves the detection probability and energy efficiency of a network through simulations.

Muchandi et al.^32^ developed a secure routing method for cognitive radio ad hoc networks. The proposed method used a cross-layer routing system with Kolmogorov-Smirnov and sequential probability tests for filtering false sensing measurements. The method guarantees privacy and QoS through adaptive queuing and source rate control, mitigating the attacks on spectrum sensing. The method improves the accuracy of spectrum sensing and the packet delivery ratio.

Wu et al.^33^ proposed a beamforming method to realize secure communications in cognitive radios. The approach is an iterative algorithm technique for RIS-assisted networks for jointly optimizing the secrecy and transmission rates of the system. The approach ensures a reduction in total transmit power with reliable performance against different/eavesdropping users. The method converts the secrecy rate constraints into the second-order cone form, after which the beamforming is optimized iteratively.

Physical layer security in CRN is administered using authentication and user authorization methods as seen from the above-disclosed methods. The precise CSI update after the channel allocation and utilization is less in most methods discussed. This precise update identifies the need for modified/ retained authentication irrespective of the number of concurrent secondary users. Besides, the authentication sequence requires mutually excluded and joint verification for different allocations and utilization from the primary and secondary user ends, respectively. Based on the authentication rate, the channel utilization is decided to retain maximum secrecy. Therefore, the CSI for the consecutive allocations is mandatory with its authentication sequence for further outage reductions. The proposed model satisfies these requirements through improved authentication blended with sequence and mutually agreed communication to reduce secrecy outages.

## Proposed secure channel estimation model (SCEM) using channel state information (CSI) and deep learning (DL)

### Introduction

**Fig. 1:**
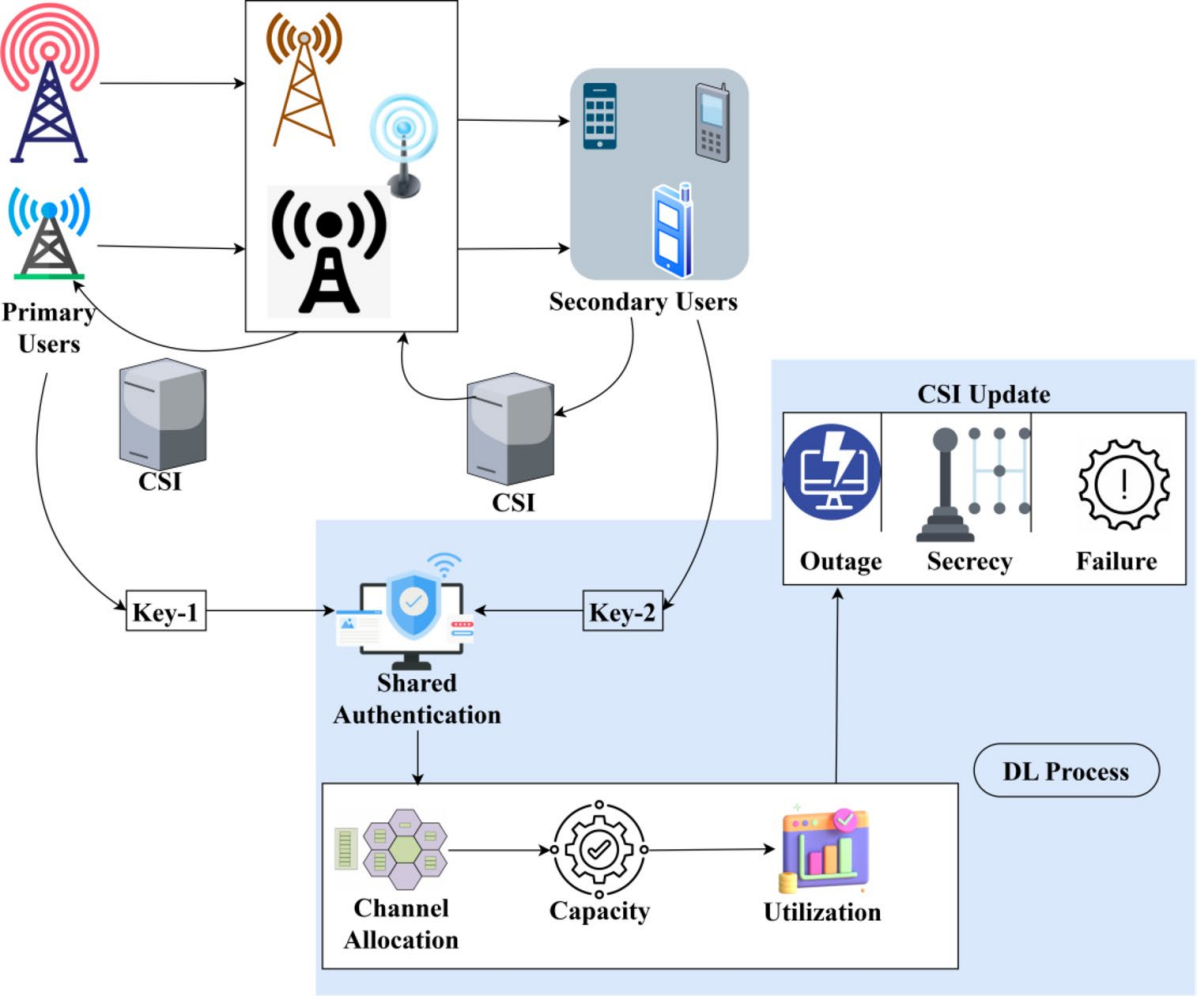
SCE model using SCI presentation.

Physical layer security is made to improve the availability, confidentiality, and integrity of the communication between the users/ devices based on primary CSI and secondary CSI inputs. The channel state information required from the various external communications (i.e.) the CSI observed from both primary and secondary users across different time instances. The objective of this model is to reduce the impact of adversaries in processing signal layers. The challenging part is the evaluation and augmentation of the channel utilization and actual channel capacity availability until the allocation intervals with the previous CSI instances. In this manner, the sequential CSI are stored as records for providing accurate security models. The proposed SCEM-CSI is presented in Fig. 1.

The channel state information of primary and secondary users is independently observed through terminals in the open environment. The two states are mainly used for external communication between the devices/ users in cognitive radio networks without interruptions or failures. In the primary channel state, the key-1 is generated and provided for primary users’ CSI authentication in different transmission intervals. Instead, in a secondary state, the key-2 is generated and provided for secondary users’ CSI authentication. A security checking and channel allocation capacity reduces the chance of eavesdroppers in the signal processing layers by causing impacts. Adversaries are detected as an instance of missing channel state information or unnecessary changes. The proposed secure channel estimation modeling focuses on such eavesdroppers in the signals processing layers through 2-level key shared authentication for improving PLS using a deep learning paradigm.

### Outage estimation

The proposed model validates two outputs (i.e.) channel capacities and utilization throughout the allocation for improving security models for sensing and beamforming. Using these outputs, the minimum and maximum eavesdroppers that occur in the signal processing layers are identified to check failed allocations based on the outage secrecy rates. Instead, if any failed allocation is identified in any transmission interval, channel capacities and utilization are changed for better operation purposes. Therefore, the two outputs are responsible for identifying the change in channel capacity and utilization that increases the security needs using deep learning without failed allocations. The capacity and utilization of the channels are estimated for checking the authentication completeness for the maximum channel capacity utilization, irrespective of adversary interference. From this proposed model, the adverse impacts in CRNs are reduced through deep learning algorithm verification that follows mutual authentication. The variables$$\:\:{P\left(U\right)}_{N}$$$$\:\:{S\left(U\right)}_{N}$$ represent the number of primary users and secondary users in CRN. The eavesdropper’s condition in signal is identified using the proposed model and thereby adversary interference is identified to prevent outage secrecy. Assume that$$\:\:{\mathbb{K}}^{1}$$ and$$\:\:{\mathbb{K}}^{2}$$ are the key-1 and key-2 used for shared authentication and thereby channel allocation$$\:\:{Chnl}_{a}$$ is analyzed to reduce adversaries. Initially, the signal processing layers$$\:\:S\left({p}_{L}\right)$$ are modeled as per Eqs. (1) and (2)

1 $$\:S\left({p}_{L}\right)=\left({Chnl}_{a}\left[{P\left(U\right)}_{N}.{\mathbb{K}}^{1}\oplus\:{S\left(U\right)}_{N}.{\mathbb{K}}^{2}\right]\right)$$

Such that,

2 $$\:\left.\begin{array}{c}\begin{array}{c}{Evd}_{i}={\sum\:}_{i=1}^{{Chnl}_{a}}{T}_{thr}-\left(1-\frac{{Sp}_{T}}{{C}_{T}}\right)\\\:\forall\:S\left({p}_{L}\right)={K}^{T}={Chnl}_{{a}_{T}}\end{array}\\\:{Sp}_{T}={Chnl}_{{a}_{T}}\:\left(or\right)\:{Sp}_{T}<{Chnl}_{{a}_{T}}\:\end{array}\right\}$$

Where,$\:\:{Evd}_{i}$ is the time to identify eavesdroppers,$\:\:{Sp}_{T}$ is the signal processing time,$\:\:{C}_{T}$ is the time for device communication and$\:\:{K}^{T}$ is the time for key generation. The variable$\:\:{Chnl}_{{a}_{T}}$ represents the channel allocation time interval using the proposed model. In this manner, the constraint$\:\:{Sp}_{T}={Chnl}_{{a}_{T}}$ leads to maximum channel capacity and utilization in different allocation intervals. The physical layer security (PLS) in CRN relies on primary and secondary users’ CSI and multiple terminals used to meet the maximum capacity channels. The adversaries are predicted through the DL process and thereby prevent eavesdroppers in the signal. The CSI is exploited to compute the channel utilization$\:\:Chnl\left(u\right)$ and actual channel capacity availability$\:\:Chnl\left(c\right)$ in the current signal processing layer. The security models for sensing$\:\:\alpha\:$ and beamforming output$\:\:\beta\:$ are taken for achieving maximum CSI exploitation using the proposed model. The outage secrecy rates are monitored and validated for maintaining consistent operation between the terminals. Therefore, the outage secrecy rate is computed based on failed allocations$\:\:Outg\left(T\right)$ is given as

3 $$\:Outg\left(T\right)={Chnl}_{a}\left(Chnl\left(u\right)+Chnl\left(c\right)-\alpha\:+\beta\:\right)$$

Such that,

4 $$\:\left.\begin{array}{c}Chnl\left(c\right)\in\:Chnl\left(u\right)=Outg\left(T\right)\\\:and,\\\:Outg\left(T\right)\in\:{K}^{T}<{Chnl}_{{a}_{T}}\end{array}\right\}$$

**Fig. 2:**
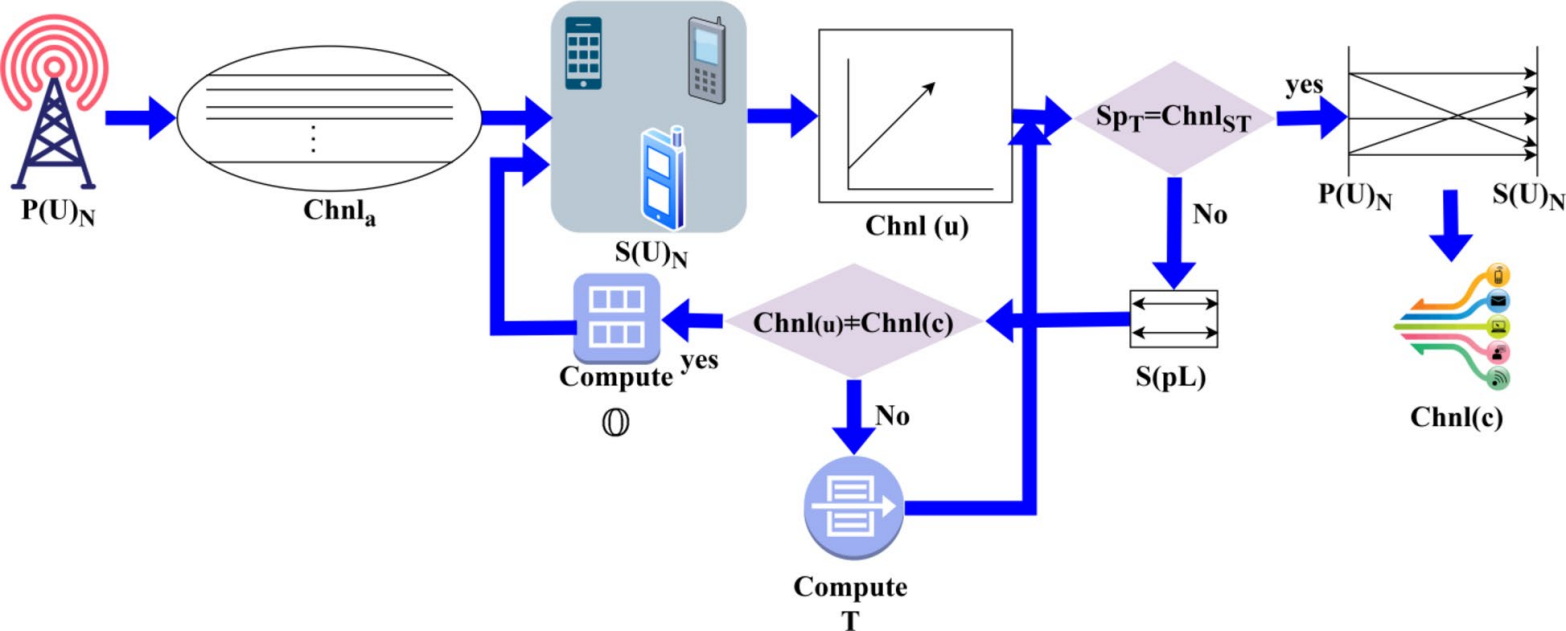
Outage identification process flow.

As per Eqs. (3) and (4), the outage and secrecy are identified in the different allocation intervals using the proposed SCEM, and the DL process is prominent for verifying the authentication completeness. The outage identification process flow is illustrated in Fig. 2.

The outage detection follows$\:\:Chn{l}_{a}$ and$\:\:Chnl\left(u\right)$ features of the$\:\:P{\left(U\right)}_{N}$ and$\:\:S{\left(U\right)}_{N}$ respectively amid the eavesdroppers. From the allocation interval,$\:\:{S}_{{p}_{T}}=Chn{l}_{{a}_{T}}$ is the balanced verification between the users to ensure lossless transmissions. This results in a stable$\:\:\beta\:$ evading$\:\:Ev{d}_{i}$ in any$\:\:{S}_{{p}_{T}}$ interval. Therefore, the alternate failing case demands the$\:\:S\left({p}_{T}\right)$ selection where$\:\:Chnl\left(u\right)\ne\:Chnl\left(c\right)$ is the$\:\:Ev{d}_{i}$ incurring condition. This results in outages across various$\:\:Chn{l}_{a}$ for that$\:\:\beta\:$ are not valid. Such intervals require a new allocation interval for various$\:\:S\left({p}_{L}\right)$ to ensure a maximum secrecy rate without interference (Refer to Fig. 2).

### Learning process

The proposed model is designed to monitor the outage and secrecy in different channel allocation intervals. The existing CSI is matched with the current CSI for similarity analysis. Based on the channel allocation, to compute accurate channel capacity and utilization for maximizing secrecy to conceal channel state information, it is prominent to follow the mutual authentication between the primary and secondary users sharing the channels with high secrecy. The transmission medium is responsible for idle CSI analysis between different users. The CSI input observed from the primary and secondary users is processed for identifying minimum and maximum CSI exploitation with high beamforming abilities. In this model, the CSI observed$\:\:\left({CSI}_{N}\right)$ for shared authentication is expressed as

5 $$\:{CSI}_{N}=\frac{\left\{{\left({Evd}_{i}\right)}_{max}-{\left({Evd}_{i}\right)}_{min}\right\}}{Outg\left(T\right)}+Chnl\left(u\right)+Chnl\left(c\right)$$

where$\:\:{\left({Evd}_{i}\right)}_{max}$ and$\:\:{\left({Evd}_{i}\right)}_{min}$ are the maximum and minimum eavesdroppers identified between the different users. The variables$\:\mathbb{\:}\mathbb{O}$,$\:\mathbb{\:}\mathbb{S},$ and$\:\mathbb{\:}\mathbb{F}$ signify the outages, secrecy, and failures identified from the current channels for improving confidentiality and integrity during device communication. That factor is not accounted for in all utilized channels. If$\:\mathbb{\:}\mathbb{O}$,$\:\mathbb{\:}\mathbb{S},$ and$\:\mathbb{\:}\mathbb{F}$ are taken place in any of the allocated channels, then increasing beamforming abilities. Now, the final DL process output for the constraints$\:\:{Chnl}_{a}>\frac{\mathbb{O}+\mathbb{S}}{\mathbb{F}}$ and$\:\:{Chnl}_{a}=\frac{\mathbb{O}+\mathbb{S}}{\mathbb{F}}$ is evaluated as in Eqs. (6) and (7). In some cases, the failures occur in channel utilization due to less integrity and confidentiality of the CSI. Therefore, these adversaries affect the$\:\:Chnl\left(c\right)$ at any intervals and from which the final DL process $\:{DL}^{T}$ is evaluated as

6 $$\:\left.\:\begin{array}{c}{DL}^{1}=Chnl{\left(c\right)}_{1}\\\:{DL}^{2}=Chnl{\left(c\right)}_{2}-{\left(\frac{\mathbb{O}+\mathbb{S}}{\mathbb{F}}\right)}_{1}-{\left(\frac{\alpha\:}{\beta\:}\right)}_{1}\\\:\begin{array}{c}{DL}^{3}=Chnl{\left(c\right)}_{3}-{\left(\frac{\mathbb{O}+\mathbb{S}}{\mathbb{F}}\right)}_{2}-{\left(\frac{\alpha\:}{\beta\:}\right)}_{2}\\\:\begin{array}{c}\vdots\\\:{DL}^{T}=Chnl{\left(c\right)}_{T}-{\left(\frac{\mathbb{O}+\mathbb{S}}{\mathbb{F}}\right)}_{T}-{\left(\frac{\alpha\:}{\beta\:}\right)}_{T-1}\end{array}\end{array}\end{array}\right\},\:{Chnl}_{a}>\frac{\mathbb{O}+\mathbb{S}}{\mathbb{F}}$$

7 $$\:\left.\begin{array}{c}{DL}^{1}=Chnl{\left(c\right)}_{1}-Evd\\\:{DL}^{2}=Chnl{\left(c\right)}_{2}-{\left(\frac{\mathbb{O}+\mathbb{S}+\mathbb{F}}{Chnl\left(u\right)}\right)}_{1}-{Evd}_{1}\\\:\begin{array}{c}{DL}^{3}=Chnl{\left(c\right)}_{3}-{\left(\frac{res*mag}{Chnl\left(u\right)}\right)}_{2}-{Evd}_{2}\\\:\vdots\\\:{DL}^{T}=Chnl{\left(c\right)}_{T}-{\left(\frac{res*mag}{Chnl\left(u\right)}\right)}_{T-1}-{Evd}_{i}\end{array}\end{array}\right\},\:{Chnl}_{a}=\frac{\mathbb{O}+\mathbb{S}}{\mathbb{F}}$$

**Fig. 3:**
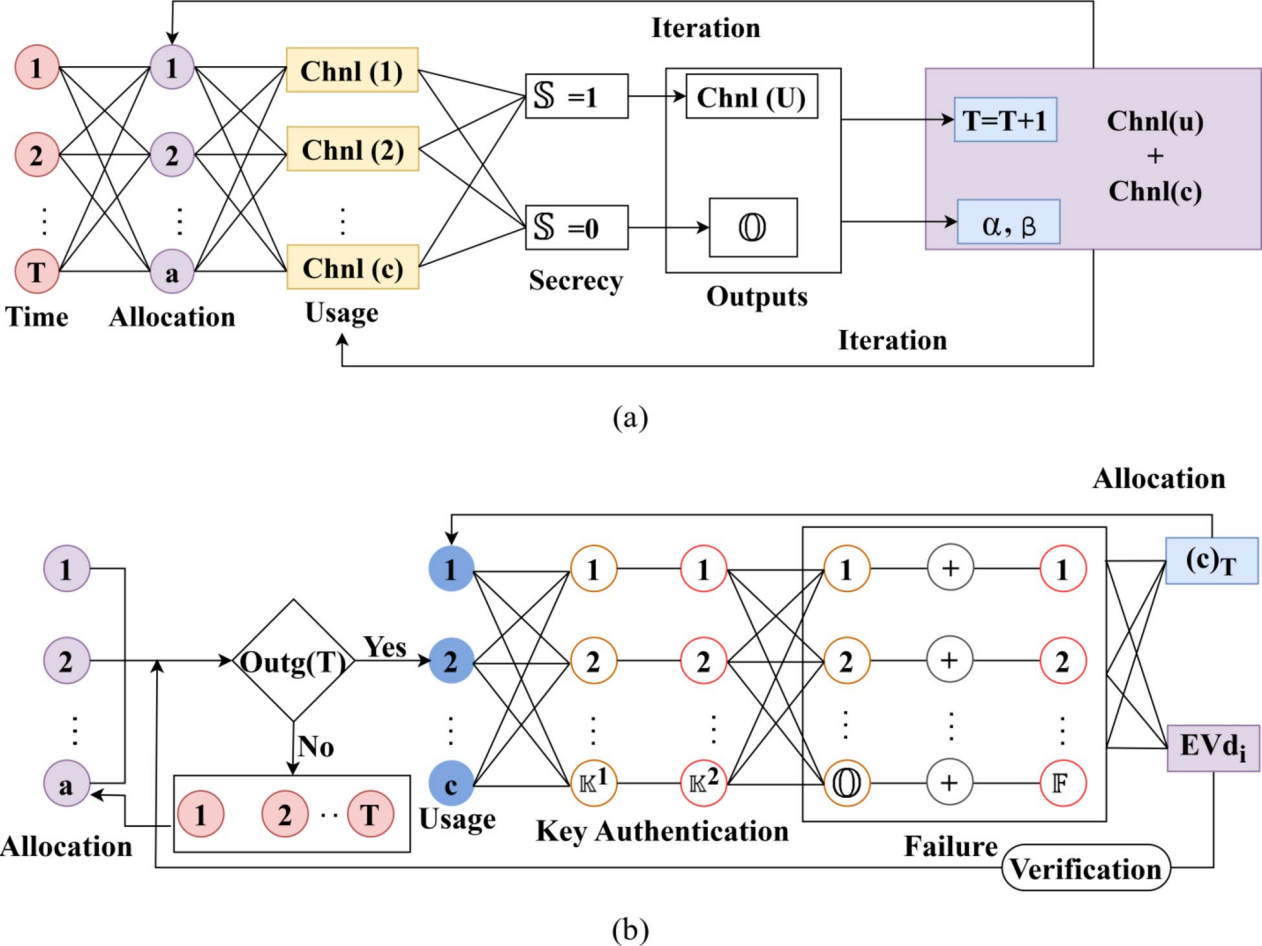
(**a**) DL model for authentication completeness. (**b**) DL model for channel utilization.

The above Eqs. (6) and (7) follows the mutual authentication of both the primary and secondary users sharing information that reduces the impact of adversaries in the signal. Here, the CSI exploitation is the uncertain condition, from which precise verification is required throughout the allocation intervals to improve the PLS. Based on $\:min$ and$\:\:max$ CSI exploitation, the early prediction of outage, secrecy, and failures are easily identified and rectified. The change in channel capacity and utilization is identified until the allocation time intervals. Authentication completeness is defined by the maximum secrecy sustained by the channel utilization and allocation regardless of the users and transmits power. This ensures the allocation, utilization, and reuses are validated through maximum secrecy. Depending on the key generation and mutual authentication processes, the authentication completeness is validated. If the utilization secrecy is retained and allocation shows up misdetections, the change in authentication is ensured. This change reverts the completeness failure for which new key is to be generated. Besides the change in authentication is similar to the integrity verification that requires multiple intervals to restore the secrecy. This verification is pursued using DL as presented in the below Figure. The channel capacity before allocation is fixed whereas the utilization varies with successful/ failed authentication. The $\:\:c$ is instantaneous for maximum users and authentication provided. Considering the $\:\:T$ and$\:\:\beta\:$ for allocation, the maximum channel utilization is achieved. In this case $\:\:{\left(c\right)}_{T}$ is the conventional allocation whereas for a failed authentication, $\:\:\left(\mathbb{O}+\mathbb{F}\right)$ is the allocation reduction. This is different (loss) compared to the conventional $\:\:Chnl\:\left(c\right)$ where$\:\:Ou{t}_{g}\left(T\right)\ne\:0$. Therefore a change in allocation is observed for failed authentications whereas$\:\:\left[Chnl{\left(c\right)}_{T}-Evd\right]$ is the actual allocation for verified authentication. The deep learning model is defined using 4 layers: one input, one output, and two conditional assessment layers. The number of neurons is equal to the $\:T$ considered for allocation, batch size$\:=\left(\frac{\mathbb{O}+\mathbb{S}}{\mathbb{F}}\right)$ and the learning rate is varied from 0.6 to 1with a minimum of 3 epochs. The loss function is modelled as a linear model that identifies the difference in allocation and utilization. Besides the components are designed as in the below illustration using the above discussion. In this case the $\:\:\left(1\:to\:T\right)$ and $\:\:Ev{d}_{i}$ iteration is pursued to minimize the difference between successive allocation. Besides, this loss function accounts the maximum utilization in the previous $\:\:T$ using the available Neurons. The DL process for authentication completeness and maximum channel capacity utilization assessment is presented in Fig. 3a and b respectively.

The inputs of DL are the time and the number of allocations in the specific time interval CSI data for $\:\:Chnl\:\left(c\right)$is integrated as a true/ false (i.e.) $\:\mathbb{\:}\mathbb{S}=1$ or$\:\mathbb{\:}\mathbb{S}=0$ case to verify authentication completeness. The allocation channel its time factor, and propagation intervals are used to define $\:\:chnl\left(u\right)+Chnl\left(c\right)$ for which the $\:\mathbb{\:}\mathbb{O}$outputs are used. The network is trained using incremented $\:\:T$ (for CSI updated intervals) and $\:\:\left(\alpha\:,\beta\:\right)$based on which new$\:\:Chnl\:\left(c\right)$ usage is defined. Therefore the DL process verifies authentication completeness to ensure fewer deviating factor occupy the$\:\:EV{d}_{i}$. The second case of validation is the $\:\mathbb{\:}\mathbb{F}$ verification irrespective of the CSI utilized. This DL process is different from the previous outputs where$\:\:Ou{t}_{g}\left(T\right)=true$ and false generate different outputs. The channel utilization assessment using DL is portrayed in Fig. 3(a). The inputs are$\:\:\left(T,a\right)\forall\:Chnl$ identified under new/previous$\:\:CS{I}_{N}$. The$\:\:Chnl\left(c\right)\forall\:\left(T*a\right)$ instances are expected to deliver$\:\mathbb{\:}\mathbb{S}=0$ is the inverse output for which$\:\mathbb{\:}\mathbb{O}$ is the constraint$\:\:\forall\:Chn{l}_{a}>\frac{\mathbb{O}+\mathbb{S}}{\mathbb{F}}$ such that$\:\mathbb{\:}\mathbb{O}=0$. Therefore, the condition$\:\:Chnl{\left(c\right)}_{T}-{\left(\frac{\mathbb{O}+\mathbb{S}}{\mathbb{F}}\right)}_{T}-{\left(\frac{\alpha\:}{\beta\:}\right)}_{T-1}$ is the deviating factor for maximum channel utilization. Following this model is the authentication completeness assessment that is presented below.

In the second DL model for authentication assessment,$\:\:{\mathbb{K}}^{1}$ and$\:\:{\mathbb{K}}^{2}$ are the balancing criteria. Depending on$\:\:c$, these$\:\mathbb{\:}\mathbb{K}$ pairs are linear provided authentication is valid under$\:\:{S}_{{p}_{T}}=Chn{l}_{{a}_{T}}$. If the authentication failure is observed in either$\:\mathbb{\:}\mathbb{O}$ or$\:\mathbb{\:}\mathbb{F}$, or both, then$\:\:{\left(c\right)}_{T}$ is least and$\:\:Ev{d}_{i}$ is high. Thus, the recurrence in verification and allocation are concurrent such that$\:\:{\left(c\right)}_{T}$ is halted to prevent any secrecy outage under$\:\:Ev{d}_{i}$. This enhances the chances of$\:\:c$ usage and$\:\:T$ distribution towards$\:\:\beta\:$ (Fig. 3(b)). The channel authentication is pursued to protect the user’s information and infrastructure using the Azure Storage Security Encryption method. In this scenario, based on the channel utilization and actual capacity evaluation, the appropriate and accurate allocation intervals are used to detect the adversaries and to improve authentication. Consistent signal transmission with high confidentiality, availability, and integrity is achieved through SCEM using CSI and DL processes to identify and segregate the minimum and maximum CSI exploitation for accurately evaluating channel capacity and utilization. The Azure Storage Security Encryption method is used to encrypt and decrypt the primary and secondary users’ CSI.

### Encryption process

The Azure storage security encryption method can perform data encryption before the information is stored and it performs data decryption to retrieve the information. This method generates keys and shares them between the primary and secondary users to ensure high confidentiality and integrity in CRN. The influencing network factors such as eavesdroppers and high-speed data transfers are supported by shared authentication in a synchronized manner. The shared key ensures end-to-end availability, confidentiality, and integrity verification for heterogeneous communication between the devices. The process of key generation and sharing differs from cluster-based outcomes. In a heterogeneous device communication, the user devices communicate with each other using CRN with PLS. Therefore, the sensing and beamforming abilities are responsible for shared authentication in a synchronized manner with less computation complexity. The synchronization is modeled for the channel utilization and capacity of keys. Therefore, the generated keys are reliable to be pursued for available users within the same communication interval. In this algorithm, the encryption and decryption process is transparently employed using 256-bit AES encryption. Based on this synchronization, the cognitive radio networks of heterogeneous environment consist of$\:\:N\left(u\right)$ number of users and$\:\:M\left(d\right)$ number of devices. The interactions between the primary and secondary users and devices are authenticated with two keys for verifying the authentication completeness with the aid of high secrecy. Let$\:\:V$ signify the key vault that stores all keys for future use. The key vault consists of different keys$\:\mathbb{\:}\mathbb{K}$. Those keys are shared for the available users to perform communication without adversary interference. Initially, the algorithm generates keys for all users communicating using CRN as denoted in Eq. (8)

8 $$\:\left.\begin{array}{c}{\mathbb{K}}^{1}={P\left(U\right)}_{N}+{S\left(U\right)}_{N}\left[\gamma\:\left({CSI}_{1}\oplus\:{Q}_{1}\right)\right]\\\:{\mathbb{K}}^{2}={P\left(U\right)}_{N}+{S\left(U\right)}_{N}\left[\gamma\:\left({CSI}_{2}\oplus\:{Q}_{2}\right)\right]\\\:\begin{array}{c}\vdots\\\:{\mathbb{K}}^{V}={P\left(U\right)}_{N}+{S\left(U\right)}_{N}\left[\gamma\:\left({CSI}_{N}\oplus\:{Q}_{N}\right)\right]\end{array}\end{array}\right\}$$

Such that

9 $$\:\left.\begin{array}{c}{fV}_{T}={\sum\:}_{i=1}^{V}{Q}_{T}-\left(1-\frac{{T}_{av}}{{CSI}_{T}}\right)\\\:\forall\:\:N\left(u\right)=V\:or\:N\left(u\right)<V\\\:and\:N\left(u\right)\in\:Communication\:of\:{fV}_{T}\end{array}\right\}$$

Where$\:\:\gamma\:(.)$ represents the non-replicative hash function,$\:\:Q$ is the random number,$\:\:{fV}_{T}$ is the time for filling the vault with different numbers of keys,$\:\:{Q}_{T}$ signifies the time for generating random integers,$\:\:{T}_{av}$ is the time for authentication verification and $\:{CSI}_{T}$ is the total time for CSI exploitation. In Eq. (9), the constraint$\:\:N\left(u\right)=V$ is satisfied for all users communicating in heterogeneous platforms in the different time instances (i.e.) the estimation and verification time leads to delivery delay$\:\:{D}_{T}>{fV}_{T}$. The primary and secondary users sharing the maximum capacity channels make use of their generated keys for high secrecy. In this model, high secrecy is imposed to prevent the anonymous changes performed over the channel state information at the time of transmission. In this algorithm, the plain text is converted into cipher text before being stored through a feasible key assignment process and the cipher text is converted into plain text for retrieval later. The primary and secondary users are eligible to communicate with the additional key vault relying on the mutual authentication label$\:\:\exists\:$ given as

10 $$\:\:\left.\begin{array}{c}\exists\:\left(N\left(u\right)\right)={Q}_{N}\left[{\mu\:}_{N}\right|\left|{\mathbb{K}}^{V}\right]\\\:for\:all\\\:\begin{array}{c}{\mu\:}_{N}\in\:N\left(u\right)\le\:V\\\:{\mathbb{K}}^{V}\in\:{fV}_{T}<{D}_{T}\end{array}\end{array}\right\}$$

**Fig. 4:**
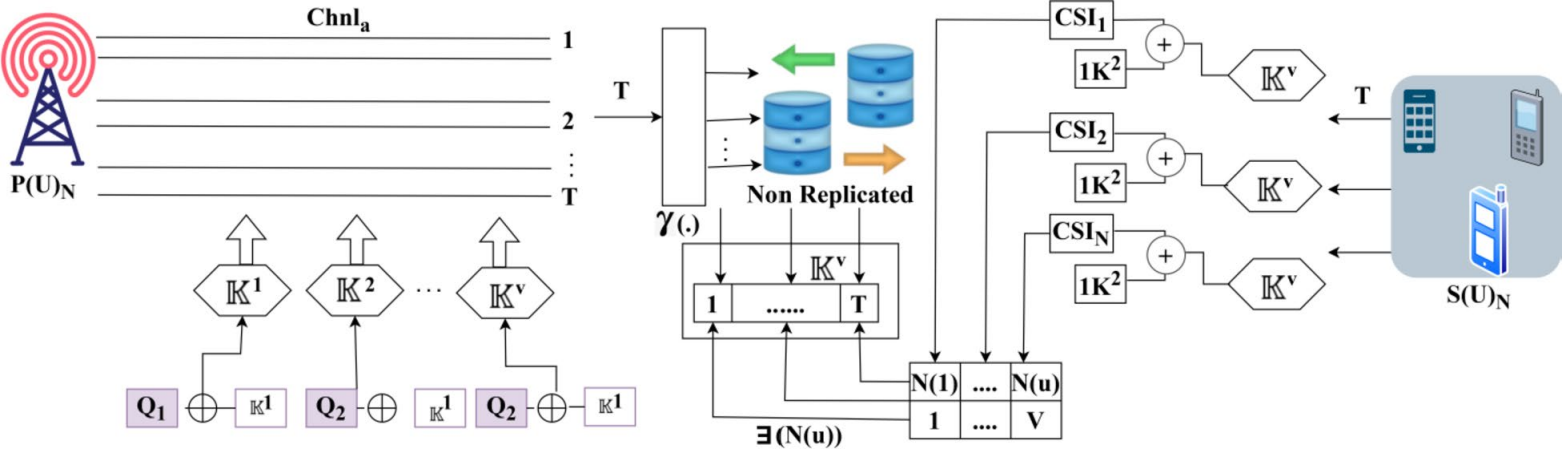
Key generation and mutual authentication processes.

In Eq. (10),$\:\:{\mu\:}_{N}$ is the user identification number, it is prominent in identifying the communicating devices/ users. In the above equation, the eligible communication channel capacity is assigned for both the primary and secondary users to replace for allocation. The key generation and mutual authentication processes are illustrated in Fig. 4.

The key generation and mutual authentication processes are detailed in Fig. 4. The authentication relies on$\:\mathbb{\:}\mathbb{K}$ assimilated using$\:\:{\mathbb{K}}^{1}$ and$\:\:{\mathbb{K}}^{2}$ in any$\:\:T$. The$\:{\mathbb{\:}\mathbb{K}}^{V}$ is an assimilation of$\:\:\left[{Q}_{N}\oplus\:{\mathbb{K}}^{1}\right]$ in$\:\:P{\left(U\right)}_{N}$ for which$\:\:\left[CS{I}_{N}\oplus\:{Q}_{N}\right]$ operates as a derivative of$\:\:f{V}_{T}$. The sequential process is instigated to ensure$\:\:N\left(u\right)=V$ (or)$\:\:N\left(u\right)<V$ is valid for any range of communication. In the continuous authentication, the$\:\:S{\left(U\right)}_{N}$ is pursued through$\:\:\uparrow\:to\:V\forall\:{\mathbb{K}}^{V}$ and$\:\:\left({\mathbb{K}}^{1}\parallel{\mathbb{K}}^{2}\right)$ under the validation$\:\:{Q}_{T}-\left(1-\frac{{T}_{av}}{CS{I}_{T}}\right)$. Hence, the number of keys generated is$\:\:{\mathbb{K}}^{V}\in\:f{V}_{T}<{D}_{T}$ with the valid constraint. Thus, the$\:\:{\mu\:}_{N}$ based authentication follows a matching 2-way$\:\:{\mathbb{K}}^{V}$ sequence to ensure high secrecy. In this mutual authentication assignment, if the constraint$\:\:N\left(u\right)<V$ is satisfied then$\:\:\left({V}_{N}\right)$. Therefore, the remaining keys are used for the successive primary and secondary users requesting communication authentication for sharing the maximum capacity channels. In this model, the key assignment process follows synchronization-based hash functions. This shared authentication is distinct for both primary and secondary users. The condition of$\:\:N\left(u\right)<V$ is modeled as a synchronized function for updating the user’s CSI based on time delay. In this condition, the process of key management and hash function is different and follows maximum CSI exploitation. The authentication completeness verification is the same for all the different channel capacity utilization, irrespective of adversary interference and time delay.

### Failed allocation verification

For the above condition, the sequence of verification is pursued unanimously for improving the PLS in CRN. In this process,$\:\:\exists\:\left(N\left(u\right)\right)$ and$\:\:\gamma\:$ are the factors that are abrupt other than the mutual authentication for the receiving CSI. Assume$\:\:\varDelta\:\left({fV}_{T}\right)$ and$\:\:\varDelta\:\left({D}_{T}\right)$ are the two functions modeled for the time that is given as

11 $$\:\varDelta\:\left({fV}_{T}\right)=\{\text{0,1}{\}}^{N\left(u\right)}=\{0,{1\}}^{V+{log}\left|V\right|-1}\forall\:N\left(u\right)=V$$

And,

12 $$\:\varDelta\:\left({D}_{T}\right)=\{{\text{0,1}\}}^{\omega\:-N\left(u\right)}\oplus\:\{\text{0,1}{\}}^{\omega\:}$$

13 $$\:=\{{\text{0,1}\}}^{\omega\:-V+log\left|\omega\:\right|-1}\oplus\:\{\text{0,1}{\}}^{\omega\:+{log}\left|\omega\:-V\right|-1}\forall\:\:N\left(u\right)>V$$

Based on the above equations, the two functions based on $\:\varDelta\:\left({fV}_{T}\right)$ and$\:\:\varDelta\:\left({D}_{T}\right)$ for the maximum capacity channels with high secrecy are satisfied. If $\:\omega\:$ and$\:\:U$ are the failed allocations and CSI updating sequence, then the mapping is pursued as, 

If

14a $$\:\left\{{CSI}_{1},{CSI}_{2},\dots\:{CSI}_{N}\right\}=\left\{{U}_{1},{U}_{2},\dots\:{U}_{N}\right\}\:\forall\:\:N\left(u\right)\le\:V$$

Else,

14b $$\begin{aligned} & \:\left\{ {CSI_{1} ,CSI_{2} , \ldots \:CSI_{{V - N\left( u \right)}} } \right\} \oplus \left\{ {N\left( u \right)_{{V - N\left( u \right) + 1}} ,\:N\left( u \right)_{{V - N\left( u \right) + 2}} , \ldots \:,N\left( u \right)_{{V - N\left( u \right) + T}} } \right\} \\ & \quad = \{ U_{1} ,U_{2} , \ldots \:,U_{{V - N\left( u \right)}} \} \oplus \{ U_{1} ,U_{2} , \ldots \:,U_{{N\left( u \right)}} \} \\ \end{aligned}$$

**Fig. 5:**
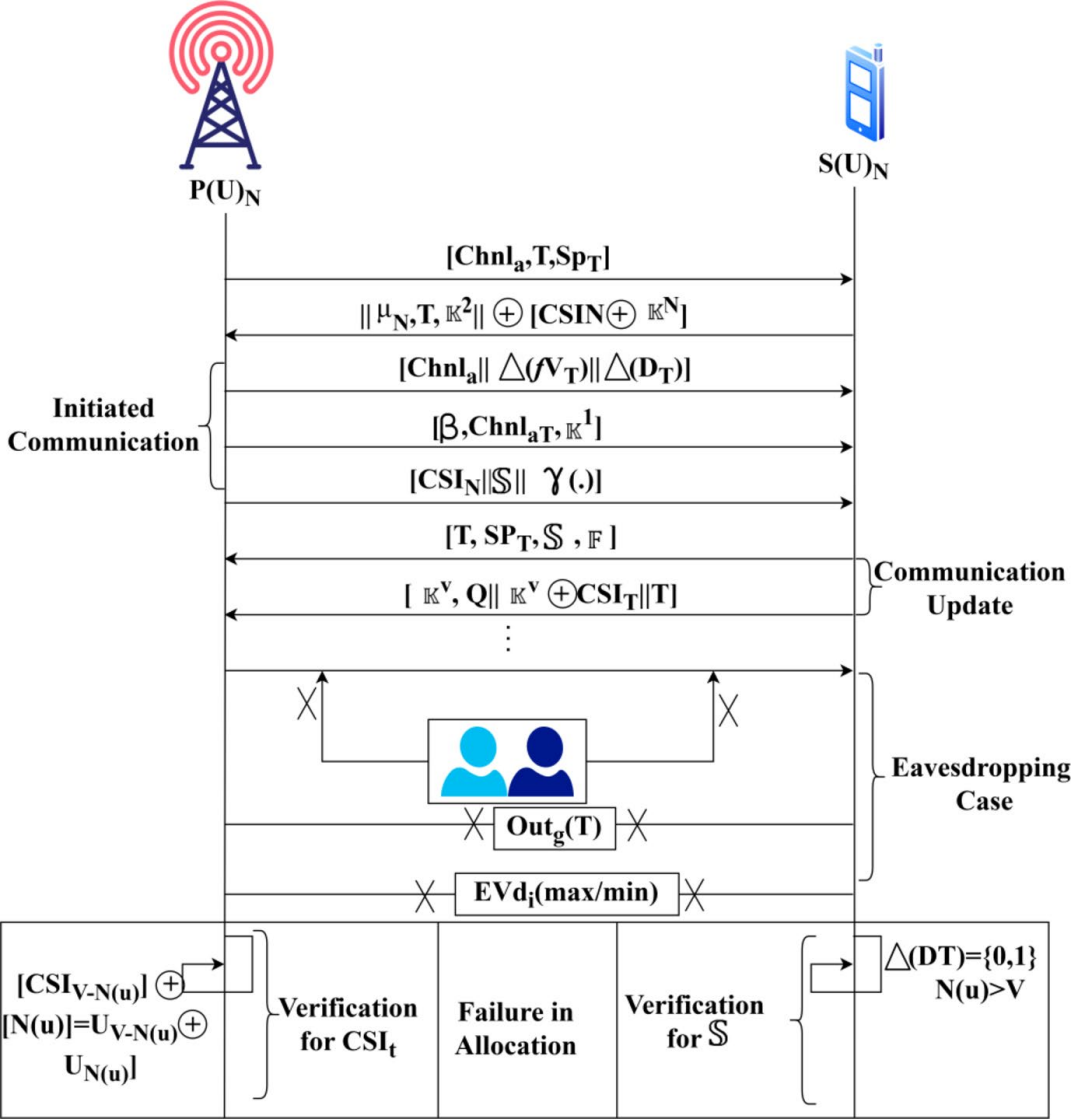
Failed allocation verification process.

In Eq. 14 (a), the CSI updates post the allocation is defined for $\:\:N\left(u\right)$and $\:\:\left[N\left(u\right)+T\right]$intervals and sequences. The communication update is presented using $\:\:\left|{\mathbb{K}}^{v},\:Q\parallel\:{\mathbb{K}}^{v}\oplus\:{CSI}_{T}\parallel\:T\right|Ch{n}_{a}|\:$ allocation at$\:\:T$. In the process$\:\:U$, the $\:\:Ou{t}_{g}\left(T\right)$ experienced results in re-authentication post$\:\:\left|{U}_{v-N\left(u\right)}+{U}_{N\left(u\right)}\right|$ verification. In Eq. (14b), the continuous sequences from $\:\:\left[1\:to\:v-N\left(u\right)\right]$ and $\:\:\left[V-N\left(u\right)+1\:to\:V-N\left(u\right)+T\right]$ is the new allocation request. The re-authentication is initiated from $\:\:\left[V-N\left(u\right)+1\right]$for which $\:\:\parallel\:T\parallel\:\forall\:\left|Chn{l}_{a}\right|$ is allocated in$\:\:\left(T+1\right)$. This is used to validate the allocation failures between $\:\:CS{I}_{N}$and $\:\:\left[N\left(u\right)\le\:V\right]$ intervals. Thus the $\:\mathbb{\:}\mathbb{S}$ verification is augmented post the break in sequence detection such that $\:\:\varDelta\:\left(DT\right)$is confined in $\:\:\left[\text{0,1}\right]$ for which re-allocation is performed. Based on the above equations, the verification process is modeled throughout the allocation intervals; the authentication verification is pursued following the two functions mentioned above. The failed allocation verification process is illustrated in Fig. 5.

The failed allocation verification is presented for 3 cases: initiated communication, communication update, and$\:\:Ev{d}_{i}$ as provided in Fig. 5. The chances of$\:\:\left[{\mathbb{K}}^{V},Q\parallel\:{\mathbb{K}}^{V}\oplus\:CS{I}_{T}\parallel\:T\right]$ is verified under$\:\:{\mu\:}_{N}$ shared$\:\:T$ for which$\:\:\left[CS{I}_{N}\oplus\:{\mathbb{K}}^{V}\right]$ is valid. In the transition between the initiated transmission and the update,$\:\:\left[Chn{l}_{a}\parallel\:\varDelta\:\left(f{V}_{T}\right)\parallel\:\varDelta\:\left({D}_{T}\right)\right]$ and it’s corresponding$\:\:\left[{\mathbb{K}}^{V},Q\parallel\:{\mathbb{K}}^{V}\oplus\:CS{I}_{N}\parallel\:\right]$ is verified. If both the cases hold under different intervals, the authentication is valid for any$\:\:T$. In the adversary case of$\:\:Ev{d}_{i}$, the minimum/maximum authentication using$\:\:\varDelta\:\left({D}_{T}\right)$ and$\:\:CS{I}_{N}$ are identified. Thus,$\:\:CSI$ at any$\:\:N\forall\:{U}_{N}$ must satisfy$\:\:N\left(u\right)=V$ and$\:\:\left[CS{I}_{V-N\left(u\right)}\oplus\:N{\left(u\right)}_{V-N}\right]=\left[{U}_{V-N\left(u\right)}\oplus\:{U}_{N\left(u\right)}\right]$ is the optimal verification condition for meeting the authentication decisions. Based on the available$\:\:T$ and$\:\:CS{I}_{N}$ updates, the$\:\:Ev{d}_{i}$ the impact is verified across multiple$\:\:\varDelta\:\left({D}_{T}\right)$ for which$\:\:\varDelta\:\left(f{V}_{T}\right)$ holds, the rest is failed. The sequence of CSI updates is performed with high beamforming abilities. If this verification exceeds the allocated time, then the time delay function is pursued. For any order of receiving the channel state information, the authentication verification is pursued under its particular class and from which no additional time or computation for analysis.

## Results and discussion

### Experimental setup

The proposed model is simulated using MATLAB considering a 100 m$\:\times\:$100m network region. The number of$\:\:P{\left(U\right)}_{N}$ is 10 and the$\:\:S{\left(U\right)}_{N}$ is 100. The transmit power is varied from 0 to 40dBm, based on the distance between$\:\:P{\left(U\right)}_{N}$ and$\:\:S{\left(U\right)}_{N}$ such that 10 m to 1200 m is the varying distance. The carrier frequency is 700 MHz and the number of channels is 32 sharing 10 MHz of the allocated frequency. In terms of security, the Azure model is used with a varying key size between 16 and 512 bits based on the user information or communication instigated. The deep learning model is trained using 2$\:\times\:$600 iterations for authentication completeness and channel utilization verification. The noise experienced in the channel is$\:\:{10}^{-4}$W and the filtering rate is 1.0 for Gaussian noise. The proposed model is analyzed using simulation based outputs for which dataset is not required. Depending on the system parameter and learning configuration, the above simulation parameters are used for performing the experiment.

### Hyperparameter analysis

The hyperparameter value-based assessments are discussed in this section using the variables defined in the above discussion. In the hyper parameter analysis the variants accounted are $\:\:D{L}^{T}\:$(100 to 1200), $\:\mathbb{\:}\mathbb{S}$(0.1 to 0.1)$\:\mathbb{\:}\mathbb{S}\mathbb{\:}rate$ (2 to 11), interference rate (o.4 to 2.8)$\:\mathbb{\:}\mathbb{K}\left(16\:to\:512\:bits\right)$. These variants are considered for monotonous and different user variants to analyse the stability and longevity. The simulation dynamics are evaluated for fixed users, varying (above) parameters and vice versa. In this analysis the hyper parameters the performance adaptable for low and high configurations are accounted to meet the real-time requirements. Besides, in the analysis, the dynamic network training parameters are induced for validating false alarm and other authentication related factors. Thus, the security and performance-centric assessments are presented below: the first performance assessment is the$\:\:Outg\left(T\right)$ based on$\:\:D{L}^{{\prime\:}}$ to$\:\:D{L}^{T}$ iteration and$\:\:\beta\:$ rates.

**Fig. 6:**
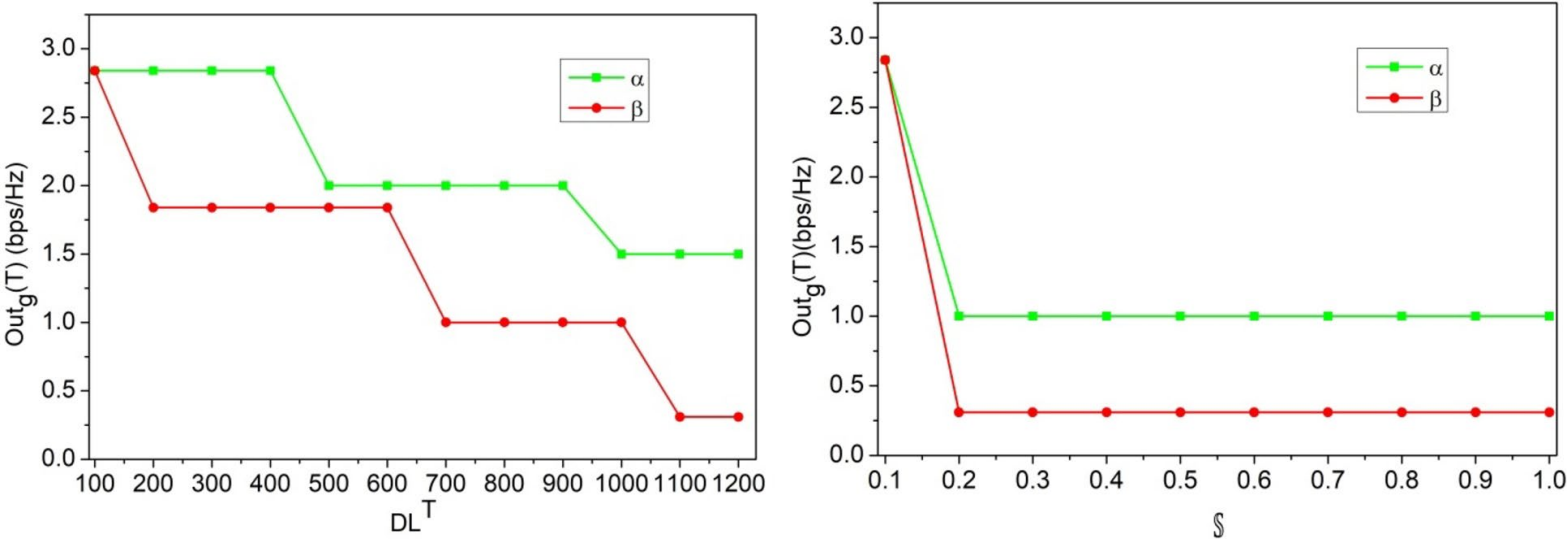
$\:\:Outg\left(T\right)$ assessment for$\:\:D{L}^{T}$ and$\:\:\beta\:$.

**Fig. 7:**
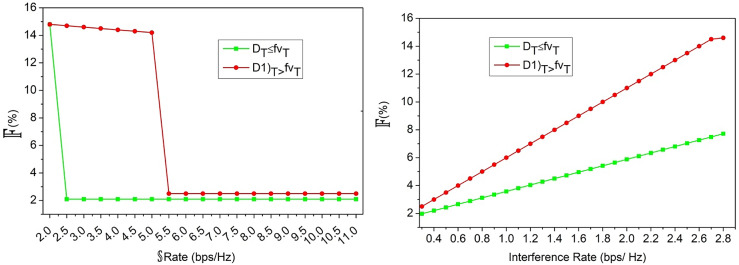
$\:\mathbb{\:}\mathbb{F}$ Assessment for$\:\mathbb{\:}\mathbb{S}$ and interference rates.

The$\:\:Outg\left(T\right)$ for the secrecy, retainment is validated in Fig. 6 for$\:\:D{L}^{T}$ and$\:\mathbb{\:}\mathbb{S}$ under$\:\:\alpha\:$ and$\:\:\beta\:$. The validation relies on$\:\:Chnl{\left(c\right)}_{T}-\left(\frac{\mathbb{O}-\mathbb{S}}{\mathbb{F}}\right)-\left(\frac{\alpha\:}{\beta\:}\right)$ provided the utilization is high. Therefore, for the maximum number of iterations, the$\:\:Chn{l}_{a}>\frac{\mathbb{O}-\mathbb{S}}{\mathbb{F}}$ and$\:\:Chn{l}_{a}=\frac{\mathbb{O}-\mathbb{S}}{\mathbb{F}}$ are the differentiating conditions for $\:\alpha\:$ and$\:\:\beta\:$ respectively. Considering the$\:\:{\mathbb{K}}^{V}\forall\:P{\left(U\right)}_{N}$ and$\:\:S{\left(U\right)}_{N}$ is utilized to ensure$\:\:f{V}_{T}$ is reliable for$\:\:\left[CS{I}_{N}\oplus\:{Q}_{N}\right]$. Therefore, the authentication case is strengthened for$\:\:{\mathbb{K}}^{V}=P{\left(U\right)}_{N}+\gamma\:\left(\bullet\:\right)$ with requiring additional sessions. Therefore, the$\:\:N\left(u\right)=f{V}_{T}$ are the surpassing conditions to maximize the affirmative sessions without$\:\:Outg\left(T\right)$. Following this, the$\:\mathbb{\:}\mathbb{F}$ parameter is analyzed under$\:\mathbb{\:}\mathbb{S}$ and varying interference rates and is presented in Fig. 7.

**Fig. 8:**
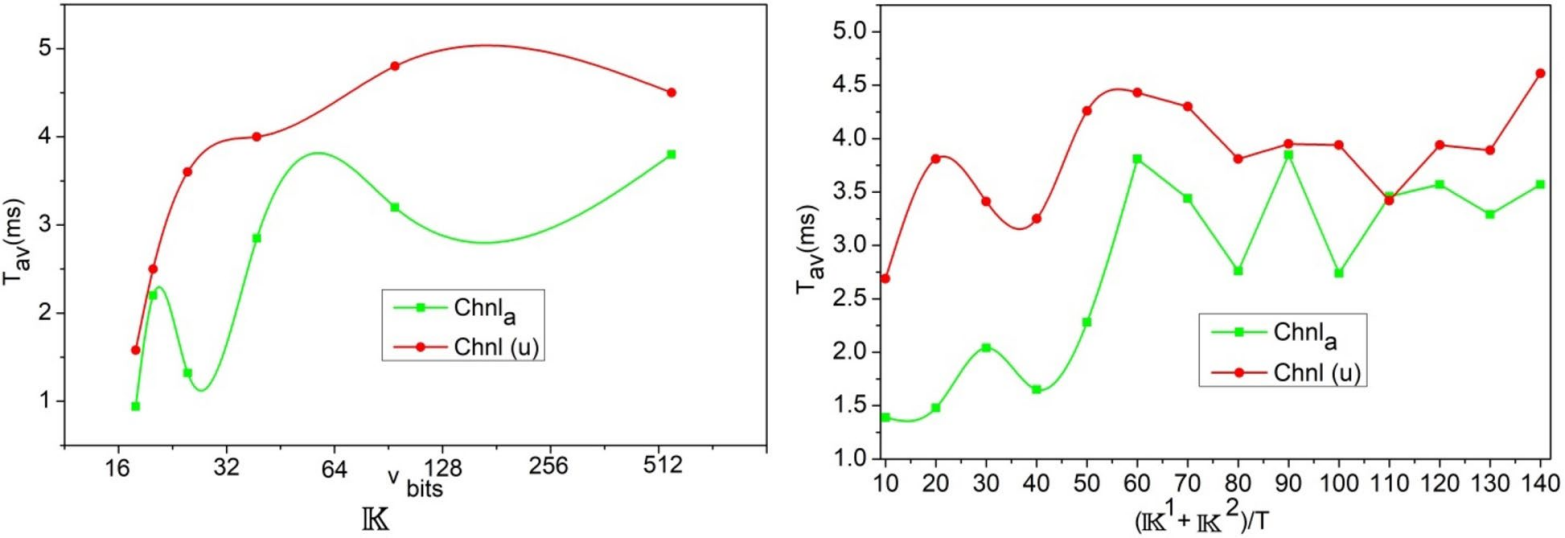
$$\:\:{T}_{aV}$$ Analysis for$$\:\:{\mathbb{K}}^{V}$$.

In Fig. 7, the$\:\mathbb{\:}\mathbb{F}$ ratio for the increasing$\:\mathbb{\:}\mathbb{S}$ and interference rates are analyzed. This analysis is presented under$\:\:\left[{D}_{T}>f{V}_{T}\right]$ and$\:\:\left[{D}_{T}\le\:f{V}_{T}\right]$ conditions. The$\:\:D{L}^{T}$ is iterated to verify if$\:\:CS{I}_{T}$ and$\:\:CS{I}_{N}$ are available post-energy communication time. Depending on$\:\:\left[Chnl\left(u\right)+Chnl\left(c\right)\right]$, the$\:\:Outg\left(T\right)\in\:{K}^{T}<Chn{l}_{aT}$ is the satisfying condition that is to be achieved. The learning model distinguishes multiple$\:\mathbb{\:}\mathbb{S}$ according to the previous$\:\:Chnl\left(u\right)$ and$\:\:Chn{l}_{a}$ concurrently. Thus, the$\:\:Outg\left(T\right)$ is confined as$\:\:\left(\beta\:-\alpha\:\right)$ for the authenticated allocations utilizing multiple capacities that are available to ensure$\:\mathbb{\:}\mathbb{F}$ is confined. Depending on these factors,$\:\:\left({D}_{T}\le\:f{V}_{T}\right)$ is suppressed under$\:\:\left[Chn{l}_{a}>\left(\mathbb{O}+\mathbb{S}\right)\right]$ and$\:\:\left[Chn{l}_{a}=\left(\mathbb{O}+\mathbb{S}\right)\right]$ respectively to ensure$\:\mathbb{\:}\mathbb{F}$ is less. Following this, the$\:\:\:{T}_{aV}$ under different$\:\:{\mathbb{K}}^{V}$ sizes and generation rates are analyzed in Fig. 8.

The$\:\:{T}_{aV}$ varies with the$\:\:{\mathbb{K}}^{V}$ bits and$\:\:\left({\mathbb{K}}^{1}+{\mathbb{K}}^{2}\right)$ increments throughout the authentication process. Using multiple allocations of$\:\:Chnl\left(u\right)$ from the various demands of$\:\:S{\left(U\right)}_{N}$, the failure verification is pursued.$\:\:\left[CS{I}_{N}\oplus\:U\left(N\right)\right]$ and$\:\:\varDelta\:\left({D}_{T}\right)=\left\{\text{0,1}\right\}$ is the range of validation pursued for each$\:\:{\mathbb{K}}^{V}$ generated. Thus, the$\:\:D{L}^{T}$ verifies the need for new$\:\:{\mathbb{K}}^{V}$ regardless of$\:\:{\mathbb{K}}^{1}$ and$\:\:{\mathbb{K}}^{2}$ generated from$\:\:P{\left(U\right)}_{N}$ and$\:\:S{\left(U\right)}_{N}$. Therefore, for any$\:\:T$, the authentication rates are validated until$\:\:\left\{CS{I}_{V-N\left(u\right)}\right\}\oplus\:\left\{N{\left(u\right)}_{V-N\left(u\right)+T}\right\}=\left\{{U}_{V-N\left(u\right)}\right\}\oplus\:\left\{{U}_{N\left(u\right)}\right\}$. Based on this equated condition, the need for new key generation/improvement is validated under$\:\:CS{I}_{N}\forall\:CS{I}_{T}$ accounting$\:\:\exists\:\left(N\left(u\right)\right)={Q}_{N}\left[{\mu\:}_{N}\parallel{K}^{V}\right]$. Thus, the authentication is pursued until maximum$\:\mathbb{\:}\mathbb{S}$ and minimum$\:\mathbb{\:}\mathbb{F}$ is achieved. Therefore, the$\:\:{T}_{aV}$ is followed by the previous$\:\:CS{I}_{N}$ in any$\:\:Chn{l}_{aT}$ for which the authentication is valid and$\:\mathbb{\:}\mathbb{F}$ is less (Fig. 8). As an extension of the above analysis, the false alarm discussion with representation is given below.

**Fig. 9:**
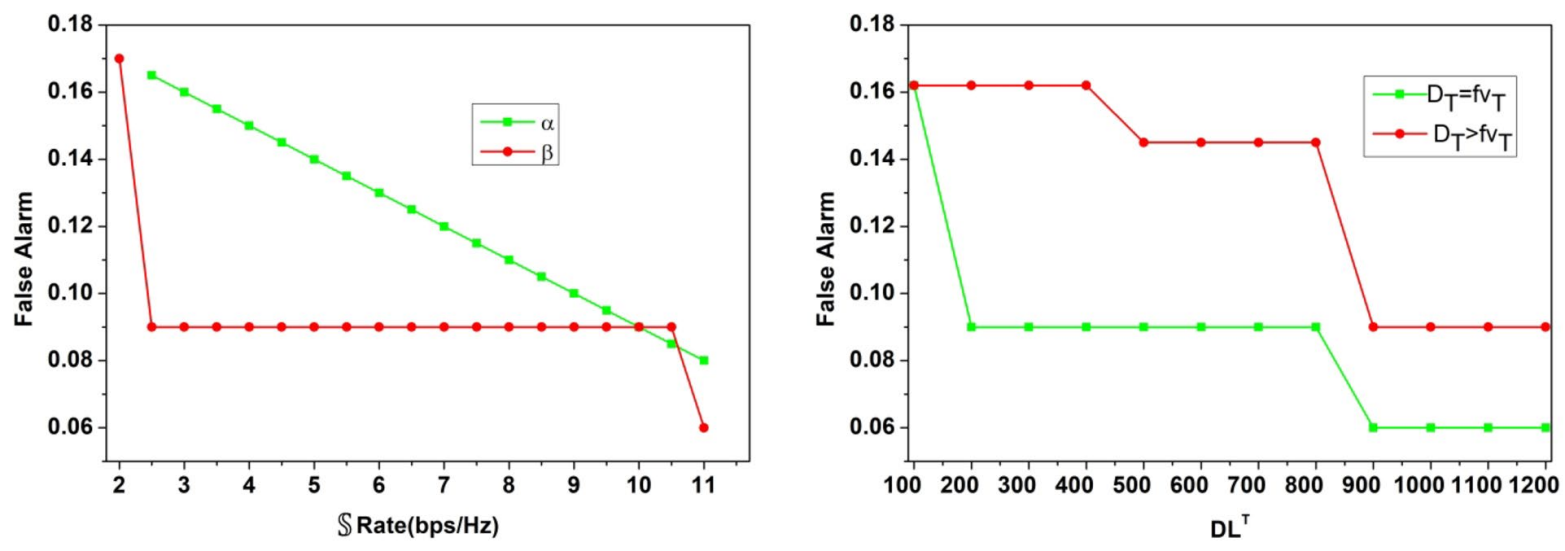
False alarm analysis.

In the above Fig. 9 analysis the false alarm for the varying0020$\:\mathbb{\:}\mathbb{S}$ rate and $\:\:D{L}^{T}$ is presented. The false rate is computed for the $\:\:\varDelta\:\left({D}_{T}\right)$ difference from$\:\:\varDelta\:\left(f{V}_{T}\right)$ is true for the maximum channel utilized. In particular, the false detection of $\:\:|{\mathbb{K}}^{v},\:\parallel\:{\mathbb{K}}^{V}\oplus\:CS{I}_{T}\parallel\:$ is regarded as the interrupted allocation under$\:\:\alpha\:$ and$\:\:\beta\:$. Compared to$\:\:\alpha\:$, the $\:\:\beta\:$ and$\:\:\left({D}_{T}=f{v}_{T}\right)$ variants reduce the false rates due$\:\:Ev{d}_{i}\left(max\right)$ other than$\:\:\varDelta\:\left(DT\right)=1\left(max\right)$. Therefore the failures in allocations are thwarted to reduce the false alarm probabilities. In the following analysis, the authentication accuracy and failures for the different variants is presented.

**Fig. 10:**
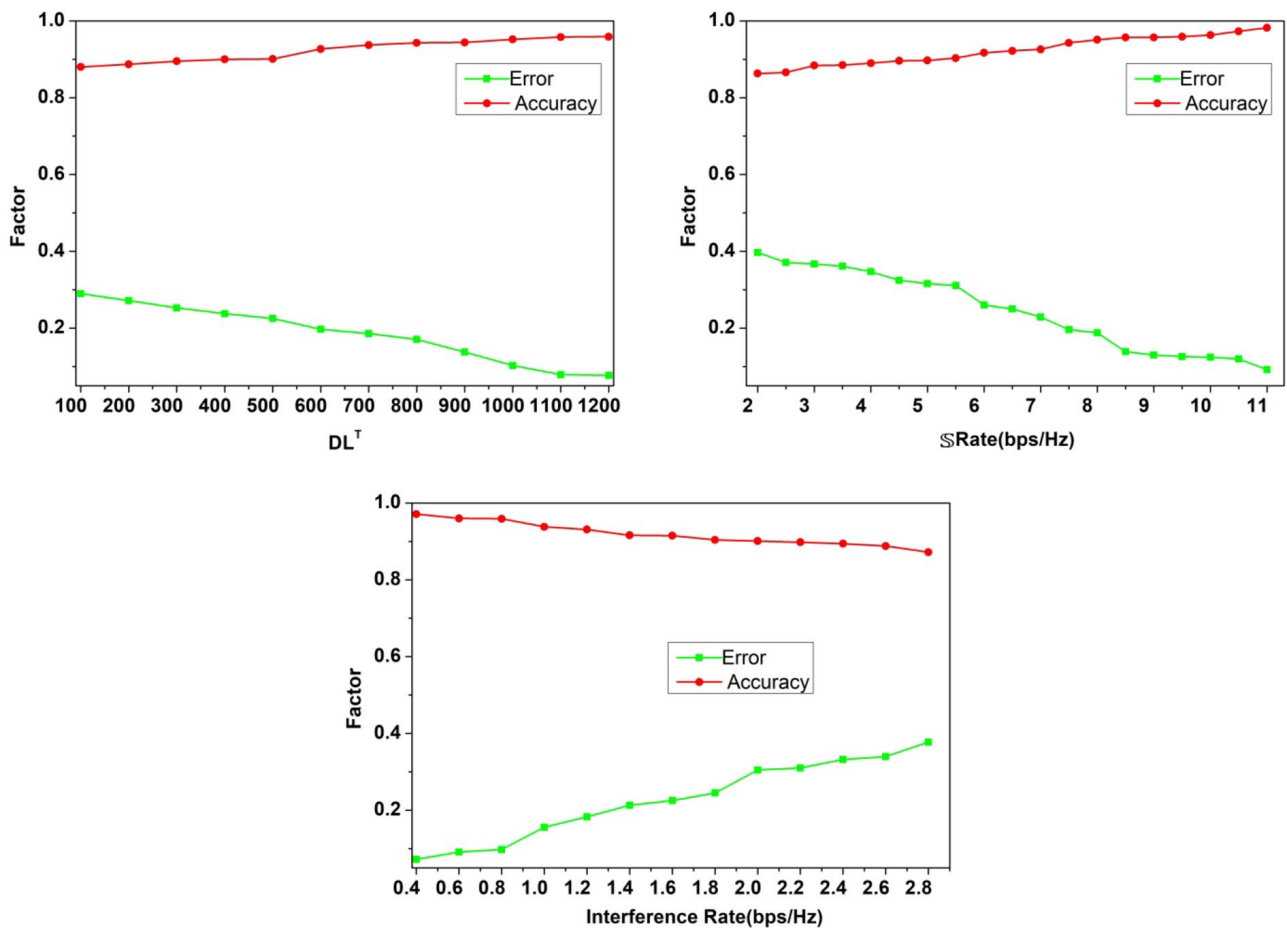
Authentication accuracy and error analysis.

The authentication accuracy and error for $\:\:D{L}^{T},\mathbb{\:}\mathbb{S}Rate,\:$and interference rate variants are analysed in Fig. 10. The training iterations and $\:\mathbb{\:}\mathbb{S}\mathbb{\:}rate$ maximizes the accuracy compared to the interference rate. In the interference identified sequences, the chances of$\:\:\left\{\right|N\left(u\right)|\ne\:|{U}_{V-N\left(u\right)}\oplus\:{U}_{N\left(u\right)}\left|\right\}\:$ is high due to which allocation failures are experienced. Through intermediate verification of $\:\varDelta\:\left(DT\right)$ and its range for new$\:\:CS{I}_{t}$, the $\:\:Ou{t}_{g}\left(T\right)$ is reduced and thereby the authentication for the consecutive interval is performed. Depending on the number of iterative verifications, the $\:\:\parallel{\mu\:}_{N},T,{\mathbb{K}}^{2}\parallel \oplus |CSIN \oplus {\mathbb{K}}^{\sim}|$ is the verifying condition to ensure maximum error less allocations. This feature ensures maximum utilization of the resources and satisfies $\:\:N\left(u\right)=V$ for high$\:\mathbb{\:}\mathbb{S}$ rates.

### Comparative analysis

The comparative analysis is performed using secrecy rate, interference, probability of missed detection, time consumption, and probability of detection metrics. These metrics are analyzed for the varying users (10–200) and the transmit power (0-40dBm). This analysis is presented as a comparative assessment along with the existing BSE-SSM^31^, BMHHO-EN^25^, JB-IA^33^, ELM-C^23^, and MA-DRL^30^ methods discussed in the related works section. The metrics used to for comparative analysis is different from the hyperparameters used. In particular, the metrics that are close to the proposed concept and the objectives are targeted for performance estimation. The channel security is ensured based on the difference between its utilization and allocation. Physical layer security is aimed to achieve high secrecy for which the metric is used. Similarly along with the signals, interference is a key factor for impacting the security and utilization. Therefore the difference between random interferences is accounted. The hyper parameter analysis presents the outage and $\:\mathbb{\:}\mathbb{F}$influenced by the maximum probability of missed detections (adversaries). This metric is inversely proportional to the adversary detection and therefore the metric is newly added. In terms of time consumption, the detection and utilization are the major concerns for which secrecy is retained. The maximum hold time is the requirement for secrecy that is impacted by the increasing users and transmits power.

#### Secrecy rate

**Fig. 11:**
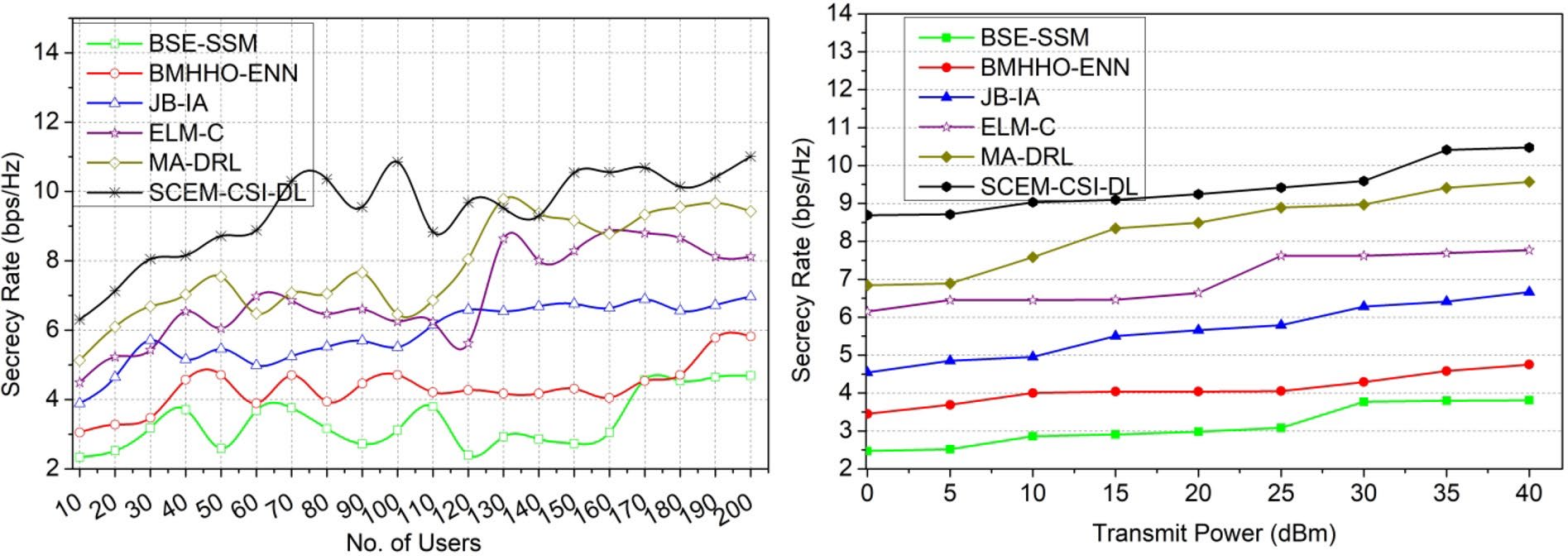
Secrecy rate comparisons.

This proposed model achieves a high secrecy rate based on verifying the authentication between heterogeneous device communications (Refer to Fig. 11). The accurate estimation of channel capacity and utilization is pursued to identify the changes. The CSI is exploited with less interference rate and time consumption is the optimal condition to improve the PLS. The outage secrecy rate is computed with channel utilization and allocation intervals for providing high confidentiality and integrity with less interference rate. This proposed model identifies the impact of adversaries on outage secrecy rate and failed allocation through the DL process. The eavesdroppers take place in the primary and secondary users sharing maximum capacity channels and are identified using the deep learning algorithm verification with a high secrecy rate. Maintaining constant transmission in specific class intervals is performed for monitoring the outages. The adversaries are identified based on identifying the changes in channel capacity and utilization with less verification time. Hence, a high secrecy rate is achieved by the current signal processing layer.

#### Interference rate

**Fig. 12:**
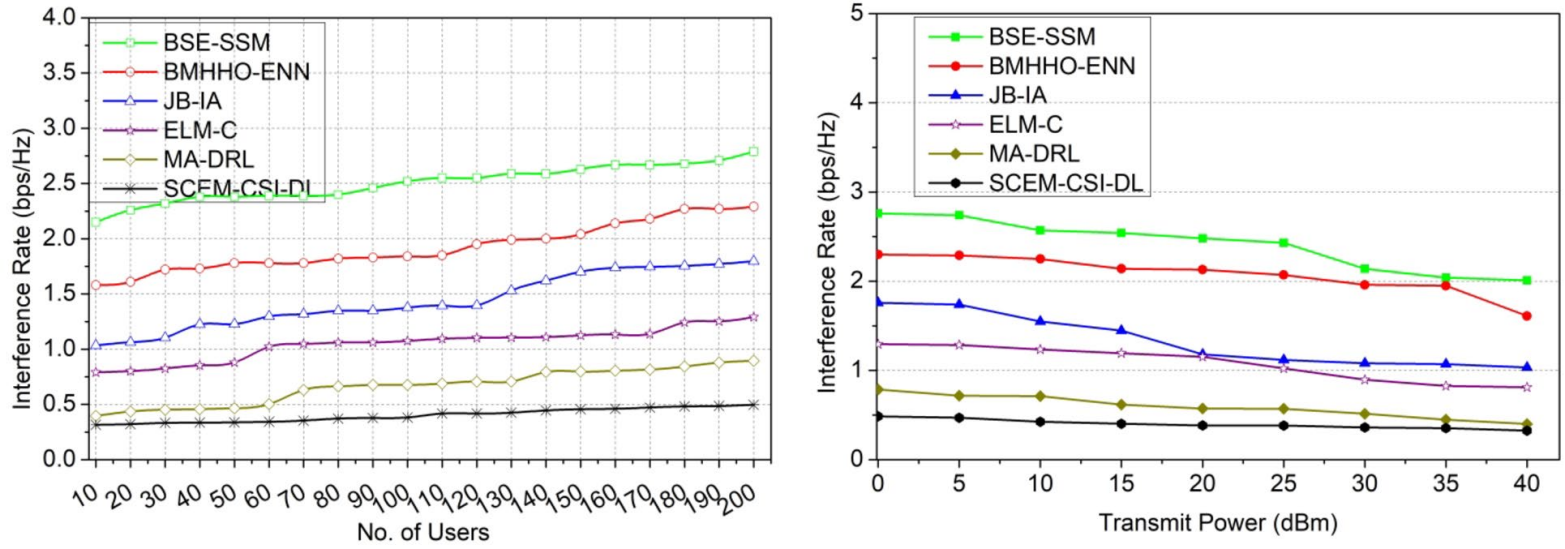
Interference rate comparisons.

The primary and secondary users-based CSI is evaluated for the external communication between devices for analyzing channel utilization and capacity to secure sensing and beamforming. Using the estimation output, the minimum and maximum CSI exploitation is addressed independently from the current signal processing layer. The eavesdroppers are addressed to identify the failed allocations such that the replacement for allocation is performed. In this article, the variation in channel capacity and utilization due to the eavesdropper’s occurrence in signal processing between heterogeneous devices is detected through a deep learning algorithm. The channel capacity changes are addressed based on the outage secrecy rate using the condition$\:\:{Sp}_{T}={Chnl}_{{a}_{T}}$ for reducing adversary interference. The maximum or minimum changes are accurately identified with high security through the Azure Storage Security Encryption method to satisfy the maximum capacity channel. Based on the maximum CSI exploitation, the early prediction of outage, secrecy, and failure allocations are easily identified and rectified. Hence, heterogeneous device communication with less interference rate is the optimal condition (Fig. 12).

#### Probability of missed detection

**Fig. 13:**
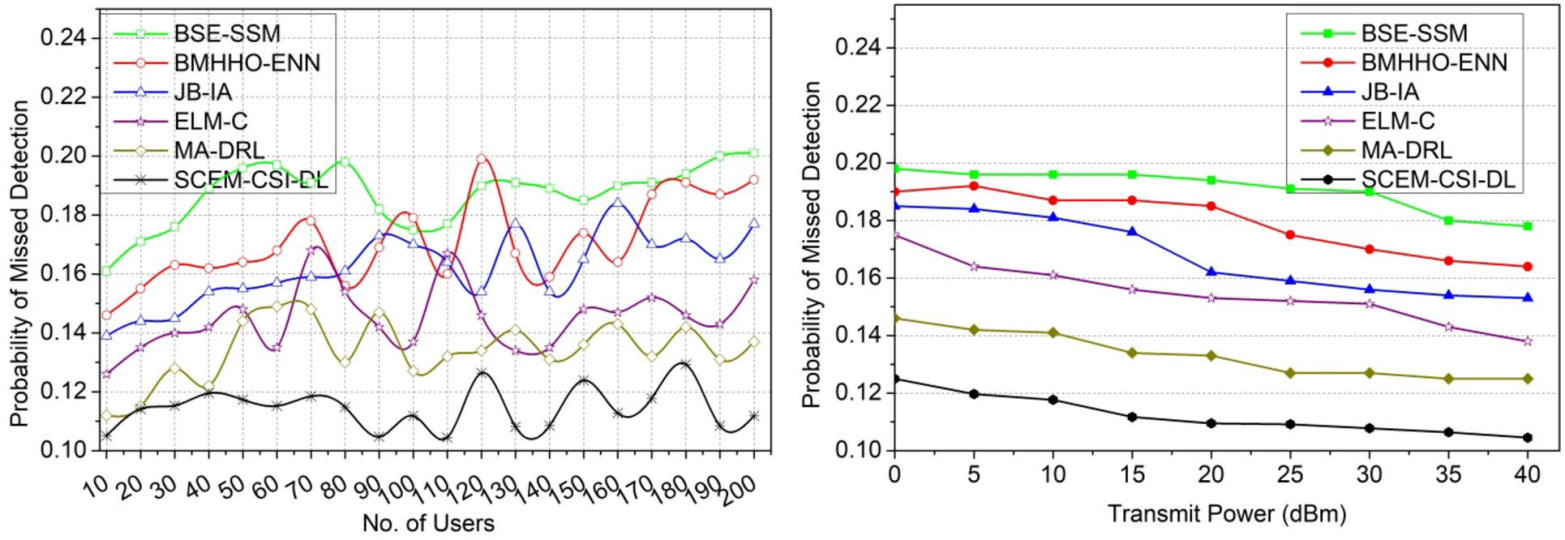
Probability of missed detection comparisons.

The channel state information is stored and retrieved securely through 2-level key shared authentication for reducing the probability of missed detection. In this model, the channel capacity, utilization, and replacement are pursued using mutual authentication and identify which user sharing maximum channel capacity utilization at the time of transmission. In this condition, the capacity and utilization variations are addressed from the verification output and thereby achieve fewer adversaries. The proposed model is employed to achieve a high secrecy rate and probability of detection for accurate channel replacement. Hence, a high secrecy rate is achievable. Based on the signal processing level, the accurate changes in channel capacity and utilization are identified using the deep learning algorithm. From the instance, the probability of missed detection is pursued to satisfy the maximum channel exploitation for reducing time consumption. In this scenario, the CSI received from external communication users/ devices is analyzed to ensure the maximum capacity channel sharing in the allocation interval. The identification of channel utilization and capacity changes with less probability of messed detection is presented in Fig. 13.

#### Time consumption

**Fig. 14:**
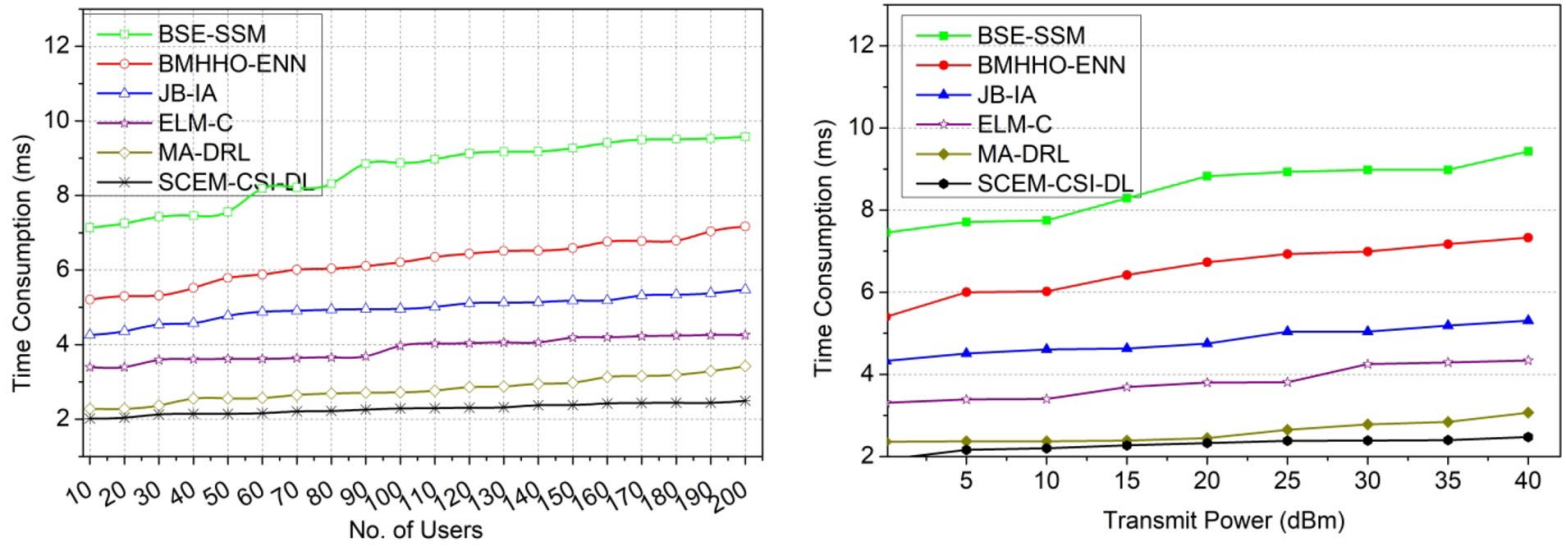
Time consumption comparisons.

The existing CSI of primary and secondary users is matched with the current for identifying the changes in capacity and utilization. Based on the channel allocation, the outages, secrecy, and failures are identified for maximum channel capacity utilization with high secrecy. To secure the channel state information before storing and before retrieving it. The CSI is prominent to follow the mutual authentication between the primary and secondary users for sharing maximum capacity channels in different intervals. The two ideal outputs are taken for precisely identifying the high or low capacity and utilization changes. Based on such adversary interference, the synchronized authentication is provided through the Azure Storage Security Encryption algorithm to repeatedly verify the outage secrecy throughout the allocation intervals. The signal processing layer is trained with shared authentication for gaining high beamforming abilities and thereby reduces capacity changes. In this scenario, the CSI exploitation based on the capacity utilization factor, the succeeding channels is taken into consideration to achieve high stability with less time consumption (Fig. 14).

#### Probability of detection

**Fig. 15:**
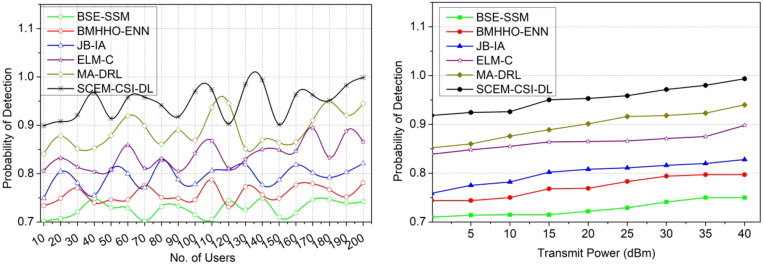
Probability of detection comparisons.

The proposed SCEM using CSI is applied to achieve a high probability of adversary detection based on addressing the changes in channel capacity utilization (Refer to Fig. 15). Here, all the information is mapped for succeeding outcomes to consider the outage secrecy rate and failed allocation with improved PLS. The PLS between different users is identified based on the actual changes and then the probability of detection towards a succeeding outcome through deep learning. Here, CSI exploitation is the uncertain condition from which reliable verification is performed for identifying changes throughout the allocation intervals. The proposed model implies the accurate replacement of allocation for the channel capacity utilization changes. The stability of the devices is computed for all the users from the current cognitive radio networks to reach the maximum CSI exploitation by using the algorithm. The eavesdroppers identified signal processing layers are replaced until the maximum capacity utilization is achieved. The trained signal processing layers are used for achieving the succeeding allocation to ensure high PLS. Hence, a high probability of detection is achievable.

### Summary

The results of the comparative analysis are tabulated in Tables 1 and 2 for the number of users and transmit power.

**Table 1:** Comparative analysis results for number of users.

Metrics	BSE-SSM	BMHHO-ENN	JB-IA	ELM-C	MA-DRL	SCEM-CSI-DL
Secrecy rate (bps/Hz)	4.69	5.82	6.97	8.12	9.43	11.011
Interference rate (bps/Hz)	2.79	2.29	1.796	1.292	0.894	0.4971
Probability of missed detection	0.201	0.192	0.177	0.158	0.137	0.1118
Time consumption (ms)	9.58	7.17	5.48	4.26	3.42	2.495
Probability of detection	0.742	0.781	0.821	0.866	0.945	0.9988

In Table 1, the comparative analysis using the existing and proposed values observed is presented. The maximum variant value (Users = 100) is used to estimate the value and the same is tabulated in the above Table. The proposed model leverages the secrecy rate by 10.38% and the probability of detection by 15.41%. This model reduces the interference rate by 10.49%, the probability of missed detection by 10.59%, and time consumption by 12.19%.

**Table 2:** Comparative analysis results for transmit power.

Metrics	BSE-SSM	BMHHO-ENN	JB-IA	ELM-C	MA-DRL	SCEM-CSI-DL
Secrecy rate (bps/Hz)	3.81	4.75	6.66	7.77	9.57	10.474
Interference rate (bps/Hz)	2.01	1.61	1.033	0.811	0.4	0.3253
Probability of missed detection	0.178	0.164	0.153	0.138	0.125	0.1045
Time consumption (ms)	9.43	7.33	5.31	4.34	3.07	2.473
Probability of detection	0.75	0.797	0.828	0.898	0.94	0.9935

In Table 2, the comparative analysis using the existing and proposed values observed is presented. The maximum variant value (Transmit Power = 40dBm) is used to estimate the value and the same is tabulated in the above Table. The proposed model leverages the secrecy rate by 10.77% and the probability of detection by 15.01%. This model reduces the interference rate by 11.07%, the probability of missed detection by 9.61%, and time consumption by 10.49%.

## Conclusion and future works

In this article, the secure channel estimation model using channel state information supported by congruent deep learning is proposed. This proposed model is designed to secure the primary and secondary user communication over eavesdroppers. The secrecy-retaining beamforming for communication is established using channel allocation and utilization differences. Based on the allocation rate, the mutual authentication between the communicating pairs is administered using a two-level key-sharing process defined by the Azure security model. The major difference between the allocation and utilization halts the authentication and key generation at any successive allocation interval. The maximum channel utilization pursues the outage secrecy verification based on interference. In the interference assessment process, the previous CSI is verified for its authentication failure and secrecy outages. Thus, the capacity utilization and authentication completeness are congruently validated using the deep learning process post the CSI update. Such a process leverages the secrecy rate by 10.38% and the probability of detection by 15.41% by reducing interference by 10.49% for the maximum number of users.

This two-level authentication requires a pre-assigned pause time between successive channel allocation intervals. This allocation prevents overlapping authentication and allocation sequences for two or more secondary users in the same beamforming range. Besides, the channel utilization with non-overlapping authentication sequences is achieved. Therefore this delay-causing problem is planned to be addressed by identifying beamforming cases before authentication with a guard interval assignment. This does not impact authentication credibility for channel utilization between any range of users.

## Data Availability

The data used to support the findings of this study have been included in this article.
